# Physical Activity in Late Prepuberty and Early Puberty Is Associated With High Bone Formation and Low Bone Resorption

**DOI:** 10.3389/fphys.2022.828508

**Published:** 2022-04-07

**Authors:** Jakob Rempe, Björn E. Rosengren, Lars Jehpsson, Per Swärd, Magnus Dencker, Magnus K. Karlsson

**Affiliations:** ^1^Department of Orthopedics, Helsingborg Hospital, Lund University, Helsingborg, Sweden; ^2^Clinical and Molecular Osteoporosis Research Unit, Clinical Sciences, Lund University, Malmo, Sweden; ^3^Department of Orthopedics, Skane University Hospital, Malmo, Sweden; ^4^Department of Physiology and Clinical Sciences, Skane University Hospital, Lund University, Malmo, Sweden

**Keywords:** bone turnover, children, puberty, physical activity, school intervention

## Abstract

**Background:**

Physical activity (PA) increases bone mass, especially in late prepuberty and early puberty, but it remains unclear if and how PA affects both bone formation and bone resorption.

**Materials and Methods:**

We included 191 boys and 158 girls aged 7.7 ± 0.6 (mean ± SD) in a population-based PA intervention study. The intervention group (123 boys and 94 girls) received daily physical education (PE) in school (40 min/day; 200 min/week) from study start and during the nine compulsory school years in Sweden. The controls (68 boys and 64 girls) received the national standard of 1–2 classes PE/week (60 min/week). During the intervention, blood samples were collected at ages 9.9 ± 0.6 (*n* = 172; all in Tanner stages 1–2) and 14.8 ± 0.8 (*n* = 146; all in Tanner stages 3–5) and after termination of the intervention at age 18.8 ± 0.3 (*n* = 93; all in Tanner stage 5) and 23.5 ± 0.7 (*n* = 152). In serum, we analyzed bone formation markers [bone-specific alkaline phosphatase (bALP), osteocalcin (OC), and N-terminal propeptide of collagen type 1 (PINP)] and bone resorption markers [C-terminal telopeptide cross links (CTX) and tartrate-resistant acid phosphatase (TRAcP 5b)]. Linear regression was used to compare age and sex-adjusted mean differences between intervention children and controls in these markers.

**Results:**

Two years after the intervention was initiated (at Tanner stages 1–2), we found higher serum levels of bALP and OC, and lower serum levels of TRAcP 5b in the intervention compared with the control group. The mean difference (95% CI) was for bALP: 13.7 (2.1, 25.3) μg/L, OC: 9.1 (0.1, 18.1) μg/L, and TRAcP 5b: −2.3 (−3.9, −0.7) U/L. At Tanner stages 3–5 and after the intervention was terminated, bone turnover markers were similar in the intervention and the control children.

**Conclusion:**

Daily school PA in the late prepubertal and early pubertal periods is associated with higher bone formation and lower bone resorption than school PA 1–2 times/week. In late pubertal and postpubertal periods, bone formation and resorption were similar. Termination of the intervention is not associated with adverse bone turnover, indicating that PA-induced bone mass benefits gained during growth may remain in adulthood.

## Introduction

Physical activity (PA) during growth induces bone mass benefits (Kannus et al., 1995; French et al., 2000; Valdimarsson et al., 2006; Alwis et al., 2008; Weaver et al., 2016). The pediatric osteoporosis prevention (POP) study, a prospective controlled study with daily school PA as an intervention, has shown that bone mass benefits may be reached on a population-based level by moderate PA (Valdimarsson et al., 2006; Alwis et al., 2008; Cronholm et al., 2020). Regular PA during growth increases peak bone mass (PBM) (Cronholm et al., 2020), defined as the highest level of bone mass in life, and high PBM is associated with high bone mass in adulthood (Tveit et al., 2015). PBM is important as it is estimated to predict 50% of the variance in bone mass at age 70 (Hui et al., 1990), and a 10% increase in PBM is expected to delay osteoporosis by 13 years (Rizzoli et al., 2010). Thus, it seems possible that high PA during growth may lead to life-long skeletal benefits.

All types of PA do not have the same osteogenic effect, and the same type of PA may have different effects during different maturational periods. Highly osteogenic PA includes dynamic activity with high loads in different skeletal directions with resting periods between exercise periods, while the number of repetitions seems of minor importance (Rubin and Lanyon, 1984; Turner and Robling, 2003; Rantalainen et al., 2010). Prospective controlled studies also suggest that PA has the greatest skeletal effect in the late prepubertal and early pubertal periods (Valdimarsson et al., 2006; Alwis et al., 2008; Cronholm et al., 2020). This notion is supported in tennis players where differences in bone mass are greater in the dominant vs. the non-dominant arm if the training was initiated before rather than after puberty (Kannus et al., 1995).

It is also important to identify whether PA-induced skeletal benefits acquired during growth remain in the long-term perspective (Karlsson et al., 2000; Tveit et al., 2015). If so, this could hypothetically reduce fracture risk later in life. Previous results from the POP study support this view, as daily school PA during the nine compulsory school years was followed by residual bone mass benefits in adulthood (Rosengren et al., 2021a,b). This view is also partly supported by others, showing that there are benefits but that these attenuate over time, raising the question of whether the benefits remain in a long-term perspective (Tveit et al., 2013a,^2015^). A reduction in PA is also reported to be followed by an increase in bone resorption markers within days (Karlsson et al., 2003). However, studies on fractures support long-term skeletal benefits, indicating that PA during growth is associated with reduced fracture risk both during growth (Fritz et al., 2016; Cöster et al., 2017) and at older ages (Tveit et al., 2013b,^2015^).

Bone turnover markers are due to the instant response to changes in PA (Karlsson et al., 2003), a possible way to increase the knowledge on the skeletal effects of PA. In adults, PA has been associated with elevated levels of bone formation markers and decreased levels of bone resorption markers (Karlsson et al., 2003). However, few studies have evaluated the effect of PA on bone turnover markers in growing children, mostly short-term and with contradicting results (Maimoun and Sultan, 2011). For example, Eliakim et al. (1997) found that in adolescent boys (Tanner stages 3–5) during a 5-week training program, bone formation markers increased and bone resorption markers decreased. In contrast, Daly et al. (1999) found no influence on bone formation in elite male gymnasts (Tanner stages 1–2) during an 18-month training period. Results from the few pediatric studies that have evaluated bone turnover markers during long periods of PA are conflicting (Banfi et al., 2010), and none have followed children with different levels of PA throughout puberty and into adult life. An overview of systematic reviews and meta-analyses regarding the effects of exercise on bone status suggested that “future studies should include bone biomarker measurements in the study design to complement radiological measurements to better understand the effects of exercise on bone” (Xu et al., 2016).

The purpose of this study was to evaluate whether children with daily school PA have higher bone formation and lower bone resorption (estimated through bone turnover markers in serum) than children with lower levels of PA, and if any residual group difference remains after the extra school PA was terminated. Our hypothesis was that children with daily PA would have high bone formation and low bone resorption, most obvious in the late prepubertal and early pubertal periods corroborating with the period where PA is known to have the greatest effect on bone mass (Karlsson et al., 2000; Tveit et al., 2015). Also, termination of daily school PA would be associated with low bone formation and high bone resorption. We specifically asked the following: do children in an intervention program with daily school PA (compared with children with regular school PA) have (i) higher bone formation and lower bone resorption, (ii) most obvious anabolic effect on bone metabolism in the late prepubertal and early pubertal periods, and (iii) lower bone formation and higher bone resorption with the termination of PA intervention?

## Materials and Methods

### Study Design

#### The Pediatric Osteoporosis Prevention Study Design

The POP study is a population-based prospective controlled intervention study with the primary aim of evaluating the effects of daily school-based PA given during the nine compulsory school years (Linden et al., 2006; Valdimarsson et al., 2006; Detter et al., 2014). We invited all children in four government-funded neighboring elementary schools to participate in this study. The students were allocated to a specific school according to their residential address. The four schools were located in southwest Malmo, Sweden, in a township with homogenous socioeconomic and ethnic structures.

#### The Intervention Design

The first school that agreed to participate also agreed to increase physical education (PE) within the school curriculum and, therefore, was assigned as an intervention school. This school increased the amount of PE from the Swedish standard of 60 min PE/week to 40 min PE/day (200 min PE/week) from the beginning of school (grade one) until the last compulsory grade (grade nine). The three remaining schools (the control group) continued with the national school curriculum of 60 min PE/week provided in 1–2 lessons/week. Since PE is a compulsory school subject in Sweden, all children had to participate according to the school schedule. In all schools, the PE lessons were led by the regular teachers who supervised a variety of activities in the ordinary PE curriculum, including ball games and activities with running, jumping, and climbing. We have no information regarding the proportions of different types of activities, intensity, duration of each activity, or if a specific student put effort into the training. We also do not have information on voluntarily chosen spare time PA beyond organized spare time PA.

#### Endpoint Variables

At each evaluation, we measured height (cm) and weight (kg) using standardized equipment. Body mass index (BMI) was calculated as weight divided by height squared (kg/m^2^). During the first 3 years, a research nurse evaluated the Tanner stage (Marshall and Tanner, 1969, 1970), and thereafter, the children reported the Tanner stage by self-assessment. We used standardized non-validated questionnaires to evaluate lifestyle (Linden et al., 2006; Valdimarsson et al., 2006; Detter et al., 2014). The questionnaires, which registered tobacco use, alcohol use, current medical conditions, medication use, exclusion of dairy products, and organized PA (weekly activity by sports clubs or sports associations) during leisure time, were completed together with parents or guardians for younger ages. Each year, headmasters reported the duration of PE classes for their school. There was no objective registration with accelerometers or force plates to measure the duration and/or intensity of the PA. We also collected blood samples from age 9.9 ± 0.6 (mean ± SD). The samples were prepared by letting the blood clot for 30 min at 8°C, followed by centrifugation at 1,430 *g* for 10 min. The serum was then stored at −70°C until analysis.

#### Participants in the Pediatric Osteoporosis Prevention Study

All children who started first grade from 1998 to 2000 in the four schools were invited to be followed up annually during their compulsory school years. Of 564 children, 349 (217 intervention and 132 control children) agreed to participate. They were then 7.7 ± 0.6 years, all were in Tanner stage 1, and 98% were of Caucasian ethnicity. We followed the children annually during the nine compulsory school years and at ages 18.8 ± 0.3 and 23.5 ± 0.7.

#### Participants in This Report

**FIGURE 1:**
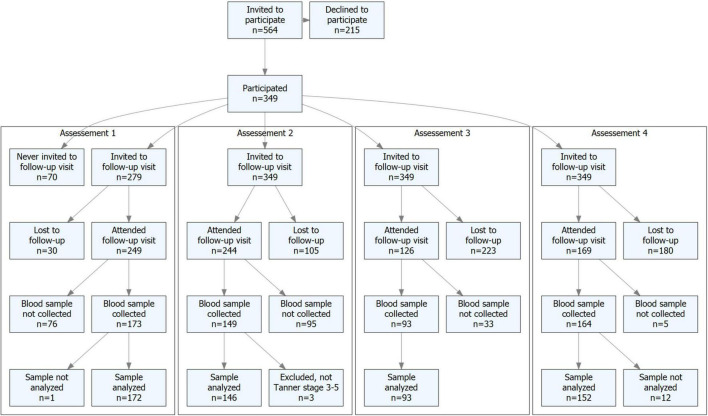
Flowchart of study participants.

In this report, we analyzed blood samples with the intervention ongoing in ages 9.9 ± 0.6 (*n* = 172, assessment 1) and 14.8 ± 0.8 (*n* = 149, assessment 2). To be included in the analyses, the children in assessment 1 had to be in Tanner stages 1–2 (defined as the late prepubertal and early pubertal periods) and in assessment 2 in Tanner stages 3–5 (defined as the late and postpubertal periods). Therefore, in assessment 2, we had to exclude one girl in the intervention group as she was still in Tanner stage 2, one girl in the intervention, and one boy in the control group with missing data on the Tanner stage (Figure 1). Thus, the analyses included 172 children in assessment 1 and 146 children in assessment 2. We also analyzed samples after the termination of the intervention, when the participants were 18.8 ± 0.3 (*n* = 93, assessment 3) and 23.5 ± 0.7 (*n* = 152, assessment 4) years, all in Tanner stage 5 (defined as the postpubertal period) (Figure 1).

### Dropout Analysis

In a previous dropout analysis at baseline, we used the compulsory medical examination at school start and found no clinically relevant differences at study start in height, weight, or BMI between children who agreed and declined to participate (Linden et al., 2006). In another previous dropout analysis at age 9.9 ± 0.6, we found no clinically relevant difference in baseline height, weight, or BMI in children who agreed and declined to participate (Dencker et al., 2006). We also undertook a new dropout analysis and found similar height, weight, and BMI at each of the assessments 1–4 between those who gave blood samples and those who did not (Supplementary Appendix 1).

### Laboratory Methods

All samples were analyzed in the same batch, with subjects’ serum from the 4 schools randomized between plates. We analyzed serum bone-specific alkaline phosphatase (bALP), osteocalcin (OC), and N-terminal propeptide of collagen type 1 (PINP) representing markers of bone formation (Szulc et al., 2000) and C-terminal telopeptide cross links (CTX) and tartrate-resistant acid phosphatase (TRAcP 5b) representing markers of bone resorption (Szulc et al., 2000). The bALP, CTX, PINP, and TRAcP 5b were assessed using the Immuno Diagnostic System-Specialty Immunoassay System (IDS-iSYS) analyzer (Pharmatest Services, Turku, Finland). Serum OC was assessed using the N-MID ELISA Assay Kit (Department of Clinical Chemistry, Skane University Hospital, Lund, Sweden). Tests were run according to the manufacturer’s instructions. The intra- and inter-CV% for bone markers assessed by Pharmatest Services, Turku, Finland were calculated by three quality control (QC) samples in each assay. The mean intra-assay CV% ranged: bALP: 0.6–1.8%; PINP: 1.4–2.7%; CTX: 0.5–7.0%; TRAcP 5b: 2.0–7.1%. The mean inter-assay CV% was bALP: 1.2%; PINP: 1.7%; CTX: 4.4%; and TRAcP 5b: 9.2%. The OC assay technical performance was assessed at the Department of Clinical Chemistry, Skane University Hospital, Lund, Sweden in a long-term follow-up of QC samples (over 1 year). The mean inter-assay CV% was 4.5%.

### Statistics and Ethics

We used IBM SPSS Statistics < *cps*:*sup* > ® < /*cps*:*sup* > version 27 for all statistical analyses. Data are presented as numbers (*n*), proportions (%), or means ± SD. Age and sex-adjusted differences in bone markers between children in the intervention and control groups were estimated by linear regression and presented as mean differences with 95% CI. We regarded a *p* < 0.05 as a statistically significant difference. Outliers were defined as points that fall 1.5 to 3 times the interquartile range above the third quartile or below the first quartile and extreme outliers as points that fall more than 3 times the interquartile range above the third quartile or below the first quartile.

This study was approved by the Ethics Committee of Lund University, Sweden (LU 453-98; 1998-09-15), conducted according to the Declaration of Helsinki, and registered as a clinical trial (Clinical Trials.gov.NCT00633828). All children and parents/guardians provided written consent before the study started.

## Results

Data on anthropometry, pubertal stage, and lifestyle characteristics are presented separately for boys (Table 1) and girls (Table 2).

**TABLE 1:** Anthropometry, pubertal development (Tanner stage), and lifestyle characteristics in the boys.

	Assessment 1		Assessment 2		Assessment 3		Assessment 4	
	Intervention	Control	Intervention	Control	Intervention	Control	Intervention	Control
Participants (n)	51	42	53	29	31	18	51	25
Age (Years)	9.9 ± 0.6	10.0 ± 0.6	14.8 ± 0.7	15.0 ± 0.8	18.8 ± 0.2	18.8 ± 0.4	23.6 ± 0.7	23.4 ± 0.5
Height (cm)	140.5 ± 7.0	141.1 ± 7.2	172.6 ± 7.9	174.7 ± 8.8	182.0 ± 6.8	181.2 ± 5.9	180.4 ± 7.2	180.7 ± 6.5
Weight (kg)	35.0 ± 7.3	34.0 ± 7.5	61.2 ± 13.3	63.1 ± 13.4	77.3 ± 12.6	75.2 ± 12.3	78.9 ± 12.4	78.4 ± 10.7
Body Mass Index (kg/m^2^)	17.6 ± 2.8	16.9 ± 2.5	20.4 ± 3.4	20.6 ± 3.6	23.4 ± 4.0	22.8 ± 3.1	24.1 ± 3.0	24.0 ± 2.9
Tanner (1–2/3–4/5) (n)	51/0/0	42/0/0	0/18/35	0/10/19	0/0/31	0/0/18	0/0/51	0/0/25
Exclusion of dairy products [n (%)]	N/A	1 (2%)	0 (0%)	0 (0%)	0 (0%)	1 (6%)	3 (6%)	1 (4%)
Chronic medical conditions [n (%)]	N/A	2 (5%)	4 (8%)	1 (3%)	6 (19%)	3 (17%)	17 (33%)	5 (20%)
Eating disorders (Bulimia, anorexia) [n (%)]	N/A	0 (0%)	0 (0%)	0 (0%)	0 (0%)	0 (0%)	1 (2%)	0 (0%)
Milk intolerance [n (%)]	N/A	0 (0%)	1 (2%)	0 (0%)	1 (3%)	2 (11%)	3 (6%)	3 (12%)
Gluten intolerance [n (%)]	N/A	0 (0%)	0 (0%)	0 (0%)	0 (0%)	1 (6%)	0 (0%)	0 (0%)
Current medication [n (%)]	N/A	0 (0%)	2 (4%)	0 (0%)	0 (0%)	0 (0%)	2 (4%)	1 (4%)
Vitamin D supplements [n (%)]	N/A	N/A	N/A	N/A	1 (3%)	0 (0%)	1 (2%)	1 (4%)
Smoking [n (%)]	N/A	N/A	0 (0%)	0 (0%)	2 (6%)	5 (28%)	3 (6%)	0 (0%)
Drinking alcohol [n (%)]	N/A	N/A	6 (11%)	3 (10%)	31 (100%)	18 (100%)	49 (96%)	23 (92%)
Total organized PA (Hours/Week)	10.2 ± 4.6	5.4 ± 3.0	10.8 ± 5.1	6.4 ± 3.4	8.5 ± 7.0	4.5 ± 2.3	5.1 ± 4.4	5.2 ± 3.9

*Data are presented as absolute numbers (n) with proportions (%) or as means ± SD.*

**TABLE 2:** Anthropometry, pubertal development (Tanner stage), and lifestyle characteristics in the girls.

	Assessment 1		Assessment 2		Assessment 3		Assessment 4	
	Intervention	Control	Intervention	Control	Intervention	Control	Intervention	Control
Participants (n)	43	36	42	22	28	16	45	31
Age (Years)	9.6 ± 0.6	9.9 ± 0.6	14.7 ± 0.8	14.8 ± 0.9	18.8 ± 0.4	18.7 ± 0.3	23.6 ± 0.7	23.3 ± 0.6
Height (cm)	139.6 ± 6.1	140.4 ± 8.4	166.1 ± 5.9	165.7 ± 8.2	168.5 ± 5.3	168.3 ± 4.6	169.2 ± 5.6	167.8 ± 6.4
Weight (kg)	34.5 ± 6.6	34.4 ± 6.9	59.5 ± 10.3	55.3 ± 11.5	64.5 ± 8.9	63.1 ± 12.7	68.2 ± 11.9	63.4 ± 12.3
Body Mass Index (kg/m^2^)	17.7 ± 3.1	17.3 ± 2.2	21.5 ± 3.6	20.0 ± 3.3	22.7 ± 3.0	22.2 ± 3.6	23.9 ± 4.2	22.4 ± 3.7
Tanner (1–2/3–4/5) (n)	43/0/0	36/0/0	0/26/16	0/11/11	0/0/28	0/0/16	0/0/45	0/0/31
Exclusion of dairy products [n (%)]	N/A	0 (0%)	0 (0%)	0 (0%)	3 (11%)	0 (0%)	4 (9%)	3 (10%)
Chronic medical conditions [n (%)]	N/A	0 (0%)	5 (12%)	0 (0%)	8 (29%)	0 (0%)	16 (36%)	10 (32%)
Eating disorders (Bulimia, anorexia) [n (%)]	N/A	0 (0%)	0 (0%)	0 (0%)	0 (0%)	0 (0%)	4 (9%)	2 (6%)
Milk intolerance [n (%)]	N/A	0 (0%)	0 (0%)	0 (0%)	2 (7%)	0 (0%)	1 (2%)	3 (10%)
Gluten intolerance [n (%)]	N/A	0 (0%	0 (0%)	2 (9%)	0 (0%)	0 (0%)	1 (2%)	1 (3%)
Current medication, including birth control pills [n (%)]	N/A	0 (0%)	1 (2%)	0 (0%)	13 (46%)	9 (56%)	20 (44%)	20 (65%)
Vitamin D supplements [n (%)]	N/A	N/A	N/A	N/A	0 (0%)	0 (0%)	2 (4%)	1 (3%)
Smoking [n (%)]	N/A	N/A	3 (7%)	3 (14%)	7 (25%)	3 (19%)	7 (16%)	5 (16%)
Drinking alcohol [n (%)]	N/A	N/A	2 (5%)	3 (14%)	24 (86%)	14 (88%)	43 (96%)	30 (97%)
Total organized PA (Hours/Week)	7.2 ± 3.0	4.2 ± 2.5	9.6 ± 4.0	5.8 ± 2.3	4.5 ± 2.7	4.5 ± 3.4	5.6 ± 5.5	4.8 ± 2.4

*Data are presented as absolute numbers (n) with proportions (%) or as means ± SD.*

**FIGURE 2:**
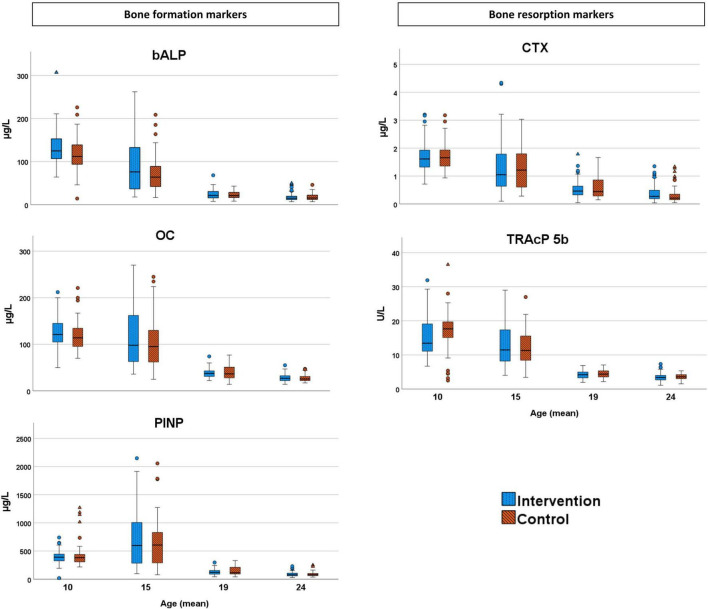
Bone formation markers [bone-specific alkaline phosphatase (bALP), osteocalcin (OC), and N-terminal propeptide of collagen type 1 (PINP)] and bone resorption markers [C-terminal telopeptide cross links (CTX) and tartrate-resistant acid phosphatase (TRAcP 5b)] in the intervention and control group. Outliers are marked as circles and extreme outliers as triangles.

With ongoing intervention in the late prepubertal and early pubertal periods (assessment 1, 2 years after the intervention was initiated), we found higher serum levels of bALP and OC and lower serum levels of TRAcP 5b in the intervention compared with the control group. The mean difference (95% CI) was for bALP: 13.7 (2.1, 25.3) μg/L, OC: 9.1 (0.1, 18.1) μg/L, and TRAcP 5b: −2.3 (−3.9, −0.7) U/L (Table 3). We found no statistically significant group differences with ongoing intervention in the late and postpubertal periods (assessment 2) or after the intervention terminated (assessments 3 and 4) (Table 3). Outliers and extreme outliers are presented in a boxplot (Figure 2). Group differences in the late prepubertal and early pubertal periods remained after the exclusion of extreme outliers as well as all outliers (data not shown).

**TABLE 3:** Bone formation markers [bone-specific alkaline phosphatase (bALP), osteocalcin (OC), and N-terminal propeptide of collagen type 1 (PINP)] and bone resorption markers [C-terminal telopeptide cross links (CTX) and tartrate-resistant acid phosphatase (TRAcP 5b)] in the intervention and control group.

	Assessment 1			Assessment 2			Assessment 3			Assessment 4		
	Intervention	Control	Mean difference	Intervention	Control	Mean difference	Intervention	Control	Mean difference	Intervention	Control	Mean difference
Participants (n)	94	78	−	95	51	−	59	34	−	96	56	−
Age (years)	9.8 ± 0.6	9.9 ± 0.6	−	14.7 ± 0.7	14.9 ± 0.8	−	18.8 ± 0.3	18.8 ± 0.3	−	23.6 ± 0.7	23.3 ± 0.6	−
**Bone formation markers:**												
bALP (μg/L)	130.0 ± 37.8	116.1 ± 37.6	**13.7 (2.1, 25.3)**	89.4 ± 59.2	73.9 ± 43.4	11.6 (−2.5, 25.8)	23.9 ± 11.4	22.6 ± 8.9	1.2 (−2.5, 4.9)	17.2 ± 8.0	17.7 ± 8.1	−0.5 (−3.0, 2.0)
OC (μg/L)	125.6 ± 28.9	117.3 ± 30.1	**9.1 (0.1, 18.1)**	115.5 ± 61.9	104.1 ± 57.0	7.0 (−9.7, 23.8)	38.4 ± 10.9	40.5 ± 15.5	−2.0 (−6.7, 2.6)	27.4 ± 7.6	27.5 ± 7.8	0.2 (−2.2, 2.7)
PINP (μg/L)	389.7 ± 113.0	414.1 ± 196.4	−26.6 (−73.8, 20.7)	694.0 ± 480.4	644.4 ± 451.0	8.2 (−114.9, 131.3)	129.3 ± 56.1	142.1 ± 70.8	−13.2 (−34.7, 8.3)	85.1 ± 36.3	88.4 ± 41.6	−3.3 (−15.5, 9.0)
**Bone resorption markers:**												
CTX (μg/L)	1.7 ± 0.5	1.7 ± 0.5	−0.01 (−0.2, 0.1)	1.3 ± 0.9	1.3 ± 0.8	−0.02 (−0.3, 0.2)	0.5 ± 0.3	0.6 ± 0.4	−0.1 (−0.2, 0.1)	0.4 ± 0.3	0.3 ± 0.3	0.04 (−0.05, 0.1)
TRAcP 5b (U/L)	15.0 ± 5.5	17.1 ± 5.1	−**2.3 (**−**3.9,**−**0.7)**	13.1 ± 5.9	12.3 ± 5.1	0.3 (−1.2, 1.8)	4.2 ± 1.2	4.6 ± 1.2	−0.3 (−0.8, 0.2)	3.5 ± 1.1	3.5 ± 0.9	0.01 (−0.3, 0.3)

*bALP data were missing in 1 intervention and 1 control child (assessment 1). OC data were missing in 3 interventions (1 in assessment 1, 1 in assessment 3, and 1 in assessment 4) and 7 control children (3 in assessment 1, 2 in assessment 2, and 2 in assessment 4). Data are presented as absolute numbers (n), means ± SD, or age and sex-adjusted mean difference with 95% CIs within parenthesis. Statistically significant differences are in bold text.*

## Discussion

Children with daily school PA have in the late prepubertal and early pubertal periods higher mean levels of the bone formation markers bALP and OC and lower mean levels of the bone resorption marker TRAcP 5b than children with lower levels of PA, but not in the late and postpubertal periods. Three and seven years after termination of the intervention, there was no adverse bone metabolism (low bone formation/high bone resorption) in the individuals with former daily school PA. These results strengthen the view that daily school PA during compulsory school years is associated with residual bone mass benefits in adulthood (Rosengren et al., 2021a,b).

The original aim of the POP study was not to once more show that specific bone-strengthening exercises in a voluntary program for children improves bone mass (Rubin and Lanyon, 1984; Kannus et al., 1995; French et al., 2000; Turner and Robling, 2003; Valdimarsson et al., 2006; Alwis et al., 2008; Rantalainen et al., 2010; Rizzoli et al., 2010; Tveit et al., 2015; Weaver et al., 2016; Cronholm et al., 2020). This is already known. Instead, we wanted to evaluate whether a PE program already used in school but extended to daily sessions in a population-based group of growing children (with some activities being more bone-strengthening and some less, and with some children being active in PE classes and some on lower levels) could be one approach to improve bone mass in society. When we in several publications have shown that this is possible (Linden et al., 2006; Valdimarsson et al., 2006; Alwis et al., 2008; Detter et al., 2014; Cronholm et al., 2020; Rosengren et al., 2021a,b), we wanted to explore in this study if and how increased gain in bone mass is associated with bone formation and bone resorption.

This study is the only published prospective study that has followed bone turnover markers in children from before puberty into adulthood in relation to different levels of PA. As previous pediatric studies have suggested that bone formation markers are positively correlated to the gain in bone mass (Lehtonen-Veromaa et al., 2000), our data, as well as previous POP studies (Valdimarsson et al., 2006; Alwis et al., 2008; Cronholm et al., 2020), support the thesis that daily PA is beneficial for the skeleton. This study adds knowledge by indicating increased bone formation (higher bone formation markers and lower bone resorption markers) in the group with daily school PA in the late prepubertal and early pubertal periods, the period with the greatest skeletal ability to respond to mechanical load (Blimkie et al., 1996; Valdimarsson et al., 2006; Alwis et al., 2008; Cronholm et al., 2020). In contrast, we found similar bone metabolism between intervention and control children in the late and postpubertal periods, corroborating with data showing that increased PA induces a lower skeletal response if initiated later in puberty (Kannus et al., 1995).

Another finding that may be of importance is the absolute higher values in most bone turnover markers, in both the intervention and control groups, in the late prepubertal and early pubertal periods (assessment 1) compared with the late and post-pubertal periods (assessments 2–4) (Figure 2). This indicates a high bone turnover in late prepubertal and early pubertal periods and may thus give a possible explanation as to why the skeleton is more responsive to mechanical load in late prepuberty and early puberty (Blimkie et al., 1996; Valdimarsson et al., 2006; Alwis et al., 2008; Cronholm et al., 2020). The findings of generally higher levels of bone turnover markers at younger ages and in early pubertal periods (assessment 1) compared with higher ages and late pubertal periods (assessments 2–4) indicate that comparisons in children should not be performed without considering age and pubertal stage.

In this study, we found no indications of adverse bone metabolic effects (low bone formation and/or high bone resorption) after withdrawal from the PA intervention. This provides a plausible explanation for the previously reported residual long-term bone mass benefits in individuals with daily school PA (Rosengren et al., 2021a,b). We were unable to draw causal conclusions regarding high PA in childhood and the absence of adverse effects on the bone turnover when the intervention is terminated. We cannot rule out a temporary adverse bone metabolism just after the program terminated, since we evaluated bone turnover markers 3 and 7 years after the program was terminated, in a period when a new steady-state may have been settled.

Participants in the former POP intervention group were also reported to have higher levels of PA after the program (Lahti et al., 2018), something that may have counteracted an adverse bone metabolism. We were also unable to exclude factors beyond the daily school PA that may be associated with bone turnover markers. Children in the intervention group may have developed greater knowledge of health-related issues, due to this consciously or unconsciously changing several health-related habits. For example, taking the stairs instead of the elevator, becoming more involved in spare time PA, and/or changing their nutritional intake. The fact that nutritional factors are of great importance during skeletal growth is supported by a systematic review that infers PA and calcium intake as the two lifestyle factors that with the highest level of evidence were shown to improve PBM (Weaver et al., 2016). Other factors of importance include vitamin D and dairy intake (Weaver et al., 2016). However, the number of individuals in our study that excluded dairy products and/or used vitamin D supplements were so few and with no obvious group differences, and seems of lesser importance for our conclusions.

The few published studies on bone turnover markers in children report conflicting results (Eliakim et al., 1997; Daly et al., 1999; Lehtonen-Veromaa et al., 2000; Maimoun and Sultan, 2011), possibly as they include sports with different types of mechanical load and children in different pubertal stages, both of which could influence bone turnover markers (Lehtonen-Veromaa et al., 2000; Maimoun et al., 2013; Vlachopoulos et al., 2017). Another contributing factor could be that the children in the studies were on a competitive level (Lehtonen-Veromaa et al., 2000; Maimoun et al., 2013; Vlachopoulos et al., 2017), which may create a risk of delayed pubertal and skeletal maturation due to intense exercise (Georgopoulos et al., 1999). In contrast, this POP study evaluates a population-based intervention on a non-competitive level, including a variety of activities and evaluations performed during predefined maturational stages.

Study strengths include the prospective, controlled, population-based study design, with longitudinal data from before puberty all the way to adulthood. Study limitations include the lack of bone turnover data prior to the initiation of the intervention. The small sample size and the high dropout frequency are other drawbacks that introduce risks for selection bias and type II errors, as well as an inability to conduct sex and/or Tanner stage-specific subgroup analyses. However, the dropout analyses revealed no obvious selection bias, and we compared all children together and not by sex, to reduce the risk of a type II error. An assessment just after the program was terminated, as well as a longer post-intervention follow-up period would have been beneficial as we do not know whether there were any group differences in the first week/month after termination of the program and/or if the benefits remain in older ages. Since most children were of Caucasian ethnicity, living in a middle-class area, inferences cannot immediately be transferred to other ethnic groups and/or socioeconomic settings. The lack of individual randomization is another weakness, but the schools refused this at the study start due to practical problems with schedules. Further limitations include the lack of an objectively measured amount of PA. Thus, we are unable to draw conclusions regarding differences in the intensity of PA between the groups. It is possible that another intervention with higher intensity of extra training and/or with more bone-strengthening exercises, such as jumps and resistance training, could also have resulted in group differences after Tanner stages 1–2. Another concern is the use of self-assessment in the Tanner evaluation, known to be less accurate compared with assessment by a trained nurse or physician (Diemar et al., 2021). However, the use of self-assessment has been found acceptable in girls, while boys if anything overestimates their pubertal stage (Duke et al., 1980). However, we have no indications that the self-assessment would be different in the intervention and control groups.

In conclusion, daily school PA in the late prepubertal and early pubertal periods is associated with higher bone formation and lower bone resorption than school PA 1–2 times/week, while no difference is found in the late or postpubertal periods. We found no association between the termination of daily school PA and adverse bone turnover, indicating that PA-induced bone mass benefits gained during growth may remain in adulthood.

## Data Availability Statement

The registration of data and the study was performed confidentially and according to Swedish and EU data protection rules. This study is based on sensitive individual-level data protected by the Swedish personal data act. Access to full data is available upon request from the corresponding author, given that the person that is interested to use it receives ethical vetting. However, some aggregated tables can be provided by the corresponding author upon request.

## Ethics Statement

The studies involving human participants were reviewed and approved by the Ethics Committee of Lund University, Sweden (LU 453-98; 1998-09-15). Written informed consent to participate in this study was provided by the participants’ legal guardian/next of kin.

## Author Contributions

JR and MK: conceptualization, data curation, funding acquisition, investigation, methodology, supervision, validation, and writing and review-editing. BR: conceptualization, data curation, formal analysis, investigation, methodology, and writing and review-editing. LJ: conceptualization, data curation, formal analysis, investigation, methodology, validation, and writing and review-editing. PS: data curation, formal analysis, investigation, methodology, and writing and review-editing. MD: investigation, methodology, and writing and review-editing. All authors contributed to the article and approved the submitted version.

## Conflict of Interest

The authors declare that the research was conducted in the absence of any commercial or financial relationships that could be construed as a potential conflict of interest.

## Publisher’s Note

All claims expressed in this article are solely those of the authors and do not necessarily represent those of their affiliated organizations, or those of the publisher, the editors and the reviewers. Any product that may be evaluated in this article, or claim that may be made by its manufacturer, is not guaranteed or endorsed by the publisher.
