# Supplementary material for: Physical Activity in Late Prepuberty and Early Puberty Is Associated With High Bone Formation and Low Bone Resorption
Source: Front Physiol. 2022 Apr 7;13:828508. doi: 10.3389/fphys.2022.828508 (PMC9021887; doi:10.3389/fphys.2022.828508)
Supplement: Supplementary file 1 [file Table_1.DOCX]

Appendix 1. Drop-out analyses including data from each assessment when comparing children who attended that assessment and left blood samples with those who attended the exam for other measurements but did not leave blood samples. Data are presented as absolute numbers (n) with proportions (percent) or means ± standard deviation.

|  | **Assessment 1** | | **Assessment 2** | | **Assessment 3** | | **Assessment 4** | |
| --- | --- | --- | --- | --- | --- | --- | --- | --- |
|  | Blood samples | No blood samples | Blood samples | No blood samples | Blood samples | No blood samples | Blood samples | No blood samples |
| Participants (n) | 173 | 76 | 149 | 95 | 93 | 33 | 152 | 17 |
| Boys/girls (%) | 54/46 | 62/38 | 56/44 | 54/46 | 53/47 | 52/48 | 50/50 | 41/59 |
| Age (years) | 9.9±0.6 | 9.6±0.6 | 14.8±0.8 | 15.0±0.6 | 18.8±0.3 | 18.7±0.4 | 23.5±0.7 | 23.8±1.0 |
| Height (cm) | 140.5±7.2 | 139.6±7.8 | 170±8.5 | 169.4±9.9 | 175.4±8.8 | 170.4±9.5 | 174.6±8.8 | 176.9±11.3 |
| Weight (kg) | 34.5±7.0 | 34.3±9.1 | 60.0±12.4 | 60.1±12.4 | 70.6±13.0 | 67.4±17.9 | 72.6±13.5 | 78.5±26.2 |
| Body Mass Index (kg/m^2^) | 17.4±2.7 | 17.4±3.3 | 20.7±3.5 | 20.9±3.9 | 22.9±3.4 | 23.1±5.2 | 23.7±3.5 | 24.7±6.3 |
